# A new protocol for concomitant needle aspiration biopsy and localization of solitary pulmonary nodules

**DOI:** 10.1186/s13019-015-0312-z

**Published:** 2015-07-29

**Authors:** Young Jo Sa, Jae Jun Kim, Young Du Kim, Sung Bo Sim, Seok Whan Moon

**Affiliations:** Department of Thoracic and Cardiovascular Surgery, College of Medicine, The Catholic University of Korea, 222 Banpo-daero, Seocho-gu, Seoul 137-701 Republic of Korea

**Keywords:** Pulmonary nodule, Preoperative localization, Thoracoscopy, PCNA

## Abstract

**Background:**

Pulmonary nodules may require thoracoscopic resection in cases where percutaneous needle aspiration (PCNA) is non-diagnostic or not technically feasible. We developed a new protocol to localize pulmonary nodules concomitantly with PCNA. We retrospectively reviewed the use of concomitant PCNA and preoperative localization under computed tomography (CT) guidance.

**Methods:**

From Jan 2006 to Dec 2013, we performed PCNA and localization concomitantly on 34 pulmonary nodules (in 33 patients) using self-made, platinum microcoils. Patients in which PCNA results were less likely to be non-diagnostic and who were anticipating thoracoscopy were eligible to participate in this study. The CT-guided PCNA biopsy and microcoil localization was performed on the day of the VATS in the CT suite. The PCNA specimen was sent to the pathologist for frozen section pathology. If diagnosis of the lesion was not confirmed by PCNA or was primary lung cancer, the patient was moved to the operating room for VATS surgery.

**Results:**

Between Jan 2006 and Dec 2013, concomitant PCNA and localization were successfully performed on 34 pulmonary nodules from 33 patients (one patient had two nodules). Of the 34 nodules, seven were diagnosed pathologically using PCNA, and 27 nodules that could not be diagnosed by PCNA were excised by thoracoscopic resection without additional procedures or time because of concomitant localization. There were no deaths or significant morbidities. Minor complications included three incidents of lung hemorrhage and five of pneumothorax (two required closed thoracostomy drainage). Of 34 nodules in which both PCNA and localization were used, thoracoscopic resections were performed on 33, lobectomies were performed concomitantly with thoracoscopic resection on 11. Intraoperative fluoroscopy was used to detect 33 of 34 nodules localized using the platinum microcoil (97.06 %) or to guide stapling during thoracoscopic resection.

**Conclusions:**

The advantages of this technique are 1) there is no need for further localization during thoracoscopy even in cases of unsuccessful PCNA, 2) it is more effective with respect to both cost and time, and 3) it provides greater patient comfort.

## Background

With widespread clinical use of high-resolution computed tomography (CT), numerous small pulmonary nodules are frequently detected during lung cancer screenings, follow-up examinations after cancer treatment, and during routine examinations for chronic diseases [1]. Approximately 150,000 new cases a year are diagnosed in the United States and approximately 40 ~ 50 % of these are malignant [2]. Percutaneous needle aspiration (PCNA) biopsy and transbronchial needle biopsy are well-established useful procedures but are less reliable to rule out malignancy than video-assisted thoracoscopic surgery (VATS) because of inadequate tissue samples or biopsy failure [3]. VATS is a useful minimally invasive tool but cannot always diagnose small, deep seated sub-pleural nodules, which are frequently neither visible nor palpable [4]. We devised a new protocol that will yield a definitive diagnosis from a needle aspiration biopsy and will also localize the nodule, which is very helpful for thoracoscopic pulmonary nodule resection when PCNA is non-diagnostic.

We introduce here our protocol for preoperative concomitant PCNA and solitary pulmonary nodule (SPN) localization using our microcoil and we review the pertinent literature.

## Methods

### Patients

From Jan 2006 to Dec 2013, we performed PCNA and localization concomitantly on 34 pulmonary nodules (in 33 patients) using self-made, fragmented (5 mm in length, 0.5 mm in diameter), platinum microcoils (Easimarker, Tae Woong Medical Co., Korea, NO. 0195053). Patients in which PCNA results were less likely to be non-diagnostic and who were anticipating thoracoscopy were eligible to participate in this study. We have performed preoperative CT-guided localization in VATS resection if the nodule meets one or more criteria: (1) maximum diameter of nodule 5 mm or less, (2) a distance from the outer margin of the nodule to the nearest pleural surface >5 mm, (3) low-density on CT. With the cooperation of the pulmonologist and radiologist, the plan was to perform PCNA and localization concomitantly. Patients with severe emphysematous lungs, central lesions, and lesions attached to bronchi or pulmonary vessels were excluded.

The Institutional Review Board of Yeouido St. Mary’s Hospital, The Catholic University of Korea approved retrospective data retrieval (approval No.SC15RISE0024). All patients agreed to undergo the procedure after being informed of the risks and benefits as well as the alternatives for management.

### CT-guided PCNA Biopsy and Microcoil Localization

**Fig. 1:**
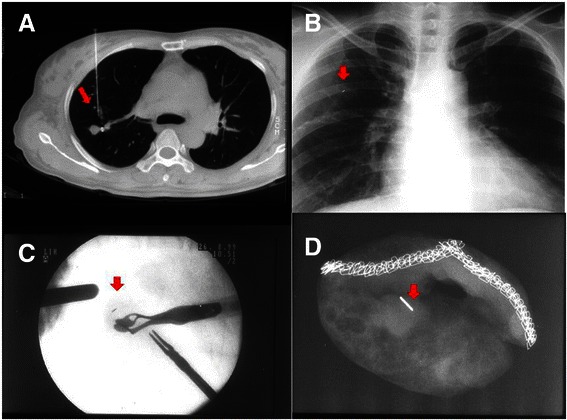
**a** Once the needle tip is confirmed to be just in contact with the nodule, the stylet is removed from the needle, and a microcoil (*arrow*) is inserted by pushing the stylet using CT guidance. After localization, PCNA (percutaneous needle aspiration) is simultaneously performed to obtain specimens for cytologic examination. **b** This chest x-ray shows the radiopacity (*arrow*) created by a microcoil inserted during PCNA. **c** During thoracoscopy, the microcoil (*arrow*) in contact with the nodule is clearly visualized on the monitor of portable fluoroscopic unit, which helps to detect the nodule and plan the stapling resection. **d** The wedge resection of the pulmonary nodule, including the microcoil (*arrow*), is identified using fluoroscopy

The CT-guided PCNA biopsy and microcoil localization was performed on the day of the VATS in the CT suite. A radiologist performed the procedure under local anesthesia. The patient was placed in a supine or prone position depending on the location of the lesion. The target lesion was detected on 1 ~ 3 mm thick axial CT sections taken on full inspiration, then punctured with a 21-gauge Chiba needle (Calibrated Chiba Stylet needle, Manan Medical Products Inc. Ill) into or just near the nodule. Once the needle tip was identified to be within the nodule or just in contact with it, the stylet was removed from the needle. Localization was performed followed by PCNA biopsy. For localization, the microcoil was completely loaded in the needle, and then introduced into the nodule or just near the nodule by pushing down the stylet. Additional CT images were routinely obtained to assess the position of the microcoil relative to the lesion and the pleural surface, and to identify any complications. If the localization failed or was inadequate, the procedure was repeated. In some cases, PCNA was performed before localization (Fig. 1). The biopsied specimen of PCNA was sent to the pathologist for frozen section pathology. If diagnosis of the lesion was primary lung cancer or not confirmed by PCNA, the patient was moved to the operating room for VATS surgery.

### Fluoroscopy-Assisted Thoracoscopic Surgery (FATS)

Thoracoscopic surgery was performed only when PCNA results were not obtained or an additional specimen was needed. Thoracoscopic surgery was performed under single lung ventilation using a double lumen endotracheal tube with three thoracoscopic ports that were made for a 5-mm or 10-mm thoracoscope, a grasper, and an endo-stapler. During thoracoscopic surgery, the fluoroscopic unit was used to detect the microcoil within or around the nodule, and to identify a generous margin for the thoracoscopic resection. Most of the needle puncture sites on the visceral pleura were identified on the thoracoscopic view, and the inserted parenchymal microcoils were clearly visualized fluoroscopically. The resected lung containing the microcoil was withdrawn into an endoscopic retrieval bag, and confirmed fluoroscopically (Fig. 1). The specimen was sent to the pathologist for frozen section pathology. If the lesion was determined to be a benign or completely resected metastatic nodule, the operation was finished. If the lesion was a non-small cell lung cancer that was surgically resectable, a lobectomy with mediastinal lymph node dissection was performed.

## Results

The study included 24 men and 9 women. The average age was 57.06 ± 10.40 (range 36 to 77) years. The diagnosis was not confirmed pathologically in all lesions, but 10 nodules (in nine patients) were suspected to be pulmonary metastases from previous malignancies. Previous malignancies included uterine cervical cancer (*n* = 1), and hepatocellular (*n* = 2), colorectal (*n* = 4) and musculoskeletal (*n* = 2) cancers. Of 34 pulmonary nodules, a pathologic diagnosis was made in seven using CT-guided PCNA to obtain the specimen. Twenty-six patients were moved from the surgery room and 27 intrapulmonary nodules (two nodules in one patient) were successfully wedge-resected and pathologies confirmed on frozen section without additional time-consuming procedures. The diameter of the nodules ranged from 3 to 34 mm with a mean 14.37 ± 7.88 mm. The distance from the outer margin of the nodule to the nearest pleural surface ranged from 3 to 31 mm with a mean of 12.15 ± 7.44 mm (Table 1). There were no deaths and no significant morbidities. Minor complications related to the PCNA and localization procedure included three incidents of lung hemorrhages and five of pneumothorax (two required mini-closed thoracostomy drainage). There was one microcoil-detection failure due to intrathoracic displacement of the microcoil during FATS. The success rate of our localization technique was 97.06 % (33/34). In one localization-failed patient, we easily performed thoracoscopic wedge resection.

**Table 1:** Patient characteristics and pulmonary nodule pathology results

Patient	Sex	Age (year)	Diameter of nodule (mm)	Depth from pleura (mm)	Histologic confirmation	Localization	Final pathology	Definite treatment
1	M	53	10	11	VATS	Success	Primary Lung cancer	Lobectomy with mediastinal lymph node dissection
2	M	57	27	5	PCNA	Success	Tuberculous granuloma	None
3	M	56	31	13	PCNA	Success	Primary Lung cancer	Lobectomy with mediastinal lymph node dissection
4	F	56	12	7	VATS	Success	Tuberculous granuloma	VATS
5	M	63	11	9	VATS	Success	Metastasis from Hepatocellular carcinoma	VATS
6	M	66	17	5	VATS	Success	Tuberculous granuloma	VATS
7	F	36	6	7	VATS	Success	Metastasis from Uterine cervix cancer	VATS
8	M	57	27	5	PCNA	Success	Primary Lung cancer	Lobectomy with mediastinal lymph node dissection
9	F	53	10	11	VATS	Success	Hamartoma	VATS
10	F	66	34	22	PCNA	Success	Primary Lung cancer	Lobectomy with mediastinal lymph node dissection
11	M	77	15	7	VATS	Success	Small cell Lung carcinoma	VATS
12	M	48	21	11	VATS	Success	Carcinoid tumor	VATS
13	M	53	21	19	VATS	Success	Granuloma	VATS
14	M	68	15	30	PCNA	Success	Primary Lung cancer	Lobectomy with mediastinal lymph node dissection
15	M	44	22	25	VATS	Success	Metastasis from Hepatocellular carcinoma	VATS
16	M	52	11	9	VATS	Success	Granuloma	VATS
17	M	49	13	16	VATS	Success	Tuberculous granuloma	VATS
18	M	39	20	7	VATS	Success	Metastasis from Musculoskeletal cancer	VATS
19	F	43	3	4	VATS	Success	Metastasis from Musculoskeletal cancer	VATS
20	M	53	10	3	VATS	Failure	Granuloma	VATS
21	M	62	21	23	PCNA	Success	Primary Lung cancer	Lobectomy with mediastinal lymph node dissection
22	M	52	13	9	VATS	Success	Granuloma	VATS
23	M	74	9	11	VATS	Success	Primary Lung cancer	VATS
24	M	67	22	9	PCNA	Success	Primary Lung cancer	Lobectomy with mediastinal lymph node dissection
25	M	67	7	5	VATS	Success	Granuloma	VATS
26	M	75	11	12	VATS	Success	Primary Lung cancer	Lobectomy with mediastinal lymph node dissection
27	F	51	7	9	VATS	Success	Granuloma	VATS
28	F	44	6	14	VATS	Success	Metastasis from Colorectal cancer	VATS
29	F	69	11	22	VATS	Success	Granuloma	VATS
30	M	67	5	31	VATS	Success	Metastasis from Colorectal cancer	VATS
31	M	61	11	9	VATS	Success	Metastasis from Colorectal cancer	VATS
32	M	54	6	7	VATS	Success	Metastasis from Colorectal cancer/Granuloma	VATS
33	M	51	9	14	VATS	Success	Granuloma	VATS

**Fig. 2:**
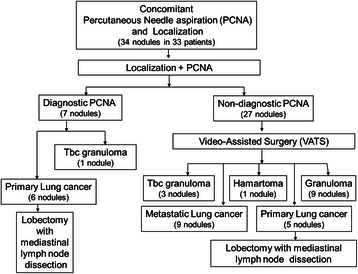
Summary of 34 nodules from 33 patients using concomitant percutaneous needle aspiration and localization protocol

Fluoroscopy assisted wedge resections were successfully performed on all 27 nodules. Histopathologic diagnoses (including diagnosis by PCNA) were successfully obtained in 34 nodules (diagnostic accuracy, 100 %) and included 11 primary cancers, nine metastatic lung cancers, nine granulomas, four tuberculous granulomas and one hamartoma (Fig. 2). All 11 patients with primary lung cancer underwent lobectomy with mediastinal lymph node dissection.

## Discussion

Improved imaging techniques such as high resolution, single breath, or spiral CT scanning, result in more frequent identification of small and often sub-centimetric SPNs [4]. Although most SPNs are benign [5, 6], the pathology of the nodule is extremely important in a patient who has previously had cancer even if the nodule is small and peripheral. In addition, even in small pulmonary lesions less than 1 cm, the overall malignancy rate is as high as, or slightly lower than that in nodules larger than 1 cm [7–9].

A PCNA biopsy is a well-established, useful procedure. However, the diagnostic accuracy of PCNA depends on the size and location of the lesion, as well as the guidance technique, and decreases from over 90 % to 25 % when the malignant nodule is small (less than 1 cm), and to 70 % when the lesion is benign [9, 10]. As many as 29 % of patients whose conditions were not diagnosed as malignant on trans-thoracic needle biopsy were ultimately found to have carcinoma [11]. Therefore, VATS has become a useful excisional biopsy tool for the diagnosis of indeterminate pulmonary nodules. Most VATS excisional biopsies are performed after obtaining a definitive diagnosis using PCNA has failed. VATS and localization are performed as separate procedures on separate days. To reduce the discomfort, cost and time of repeating localization and VATS, we have developed a simultaneous protocol that combines PCNA biopsy with localization for VATS for use in cases PCNA results were less likely to be non-diagnostic and who were anticipating thoracoscopy.

To localize small or deeply-seated difficult lesions preoperatively or intraoperatively, several techniques have been devised such as pleural marking with dyes [12] and cyanoacrylate adhesive [13], a hook-wire system [14, 15], fluoroscopy with contrast media such as barium sulfate or lipiodol [16–18], microcoils [19] or bronchoscopy-guided transbronchial metallic coils [20], and endothoracic [21] and radio-guided ultrasonography [22, 23]. Because our localization was performed with PCNA preoperatively, we had to consider pre-operative localization using a contrast media such as barium sulfate or lipiodol, hook-wire systems, and microcoils. Even if the localization material is left permanently, which can also happen when the pathologic diagnosis is obtained by PCNA, localization will not result in any problems. Contrast media injected into or around the pulmonary nodule can cause chronic inflammation in the lung (unpublished data; we observed chronic localized inflammation in our animal study). The hook-wire system also has a removal problem when the diagnosis is confirmed using PCNA. Therefore, of these methods, we selected a radiopaque wire segment, platinum microcoil, as our localization method.

In our previous study [24], we reported the results of using fluoroscopy-assisted thoracoscopy to resect small intrapulmonary lesions after preoperative computed tomography guided localization using fragmented platinum microcoils. The platinum microcoil localization was easily manipulated, did not influence the dimension or pathologic status of the nodule, and was free of a specific time frame of use before the operation. Platinum microcoils have been used as radiomarkers in stereotactic radiation treatment planning for brain tumors, head and neck tumors, uterine cervical cancer, and in marking the border or center of tumors before primary chemotherapy for breast cancer [25, 26]. The procedure can be considered so inert that no adverse side effects should be expected, even if the coils are left permanently.

With this concomitant PCNA and localization protocol, we successfully performed fluoroscopy-assisted thoracoscopic wedge resections on 27 nodules. We obtained pathologic diagnoses in all 27 nodules without another procedure although additional time (≤5 min) for localization with PCNA was necessary. This concomitant localization technique was successful in 33 of 34 nodules (97.06 %). We consider our concomitant PCNA and localization protocol to be a successful, time-saving, and efficient method for accurate diagnosis and resection of indeterminate solitary nodules.

We have performed preoperative localization if the nodule meets one or more criteria, which are (1) maximum diameter of the nodule <5 mm, (2) a distance from the outer margin of the nodule to the nearest pleural surface >5 mm, (3) low-density imaging on CT. Regarding localization criteria discussed in the literature, Saito et al. [27] demonstrated that a linear discriminant analysis using both size and depth factors (depth = 0.836 × size −2.811) might serve as an indication for preoperative localization of nodules using VATS resection. Suzuki et al. [28] considered a preoperative marking as absolutely indicated when the distance is >10 mm. According to Saito et al. criteria, 62.11 % (22/36) of the pulmonary nodules in our cases required localization; only 44.44 % (16/36) of our pulmonary nodules met the criteria of Suzuki et al. Further studies are needed to establish conditions requiring VATS, and possible conditions to localize concomitantly with PCNA to diagnose pulmonary nodules in particular. Although additional studies are needed, we think that our technique saves the merits of PCNA and VATS procedure harmoniously for diagnosing and treating the pulmonary nodules. Because there will be no need for another thoracoscopic CT localization, the cost-benefits of using this procedure can be demonstrated in labor savings, reduced CT scanning and film costs, decreased use of the localizing needle, and shorter hospital stays for patients.

## Conclusions

We conclude that concomitant PCNA and localization is the preferred technique to establish a definite diagnosis when PCNA results were less likely to be non-diagnostic and in who were anticipating thoracoscopy.
